# Assessment of particulate embolic agent distribution comparing two delivery techniques in a porcine model

**DOI:** 10.1186/s42155-026-00691-x

**Published:** 2026-04-18

**Authors:** Camilo Barragan-Leal, Ziv Haskal, Sebastian Mafeld, Dheeraj K. Rajan

**Affiliations:** 1 Joint Department of Medical Imaging, Vascular and Interventional Radiology, University Health Network / Mount Sinai Hospital, University of Toronto, Toronto, Canada; 2 Department of Radiology and Medical Imaging/Interventional Radiology. Professor of Radiology. , University of Virginia School of Medicine. , Charlottesville, USA

**Keywords:** Embolization, MicroCT, Histology, Blood vessel, Interventional Radiology

## Abstract

**Objective:**

To compare continuous vs pulsed transarterial embolization techniques with microCT and determine their impact on distal vascular penetration.

**Material and methods:**

Transarterial embolization was performed in 7 swine using 75–100-micron microspheres. In each subject, 1 kidney was embolized using continuous injection and contralateral with a pulsed injection technique. Histological analysis validated MicroCT findings (50–100 pixels, postprocedural quantification) in 2 subjects (4 kidneys), after which MicroCT alone was used for outcome assessments.

**Results:**

MicroCT imaging demonstrated similar outcomes to histology. Larger number of particles delivered with continuous vs pulsed technique (6806 vs 6174 particles into 223 ml vs 168 ml renal volume respectively). Percentage of the embolized renal cortex at 50 pixels was 1.1% for the continuous technique (0.1 – 2.3%) vs 0.7% pulsed (0.5 – 1.1%) (p = 0.3). Embolized renal cortex at 100 pixels was 2.9% continuous (1.3 – 4.2%) vs 2.3% pulsed (1.3 – 3.2%) (p = 0.3). Average kidney volume in both groups was 78.1 ml (r, 49–138) and 81 ml (r, 46–111), respectively. A slightly larger amount of embolic was delivered using the continuous vs pulsed: 14 ml (r, 12–20) vs 13.1 ml (r, 8–22). Mean embolic/kidney volume ratio was 0.20 for continuous vs 0.16 for pulsed (25% difference, p = 0.2).

**Conclusion:**

Continuous embolization technique resulted in a larger absolute concentration of embolic particles within the distal renal cortex and overall volume compared to the pulsed technique, though the difference was not statistically significant. Our findings suggest embolic delivery technique for particle embolics may influence amount and efficacy of distal embolization.

## Introduction

Despite 50 years of vascular embolization, there remains no consistent technique for injecting particulate embolics. Embolic and contrast mixtures can be delivered with short, pulsed injections or slow continuous injections [1]. However, the most effective technique for optimally filling the target vasculature to distal penetration with an embolic agent remains unclear.

Poiseuille’s law states that flow in a cylinder remains laminar until disrupted, after which it becomes turbulent [2]. Pulsed injection of an embolic solution might similarly alter flow from laminar to turbulent by periodic increases in intravascular pressure and added volume. Mechanical science literature suggests intentional pulsing can increase turbulence with alternations in fluid dispersion characteristics [3]. In humans, particle behavior and distribution may be crucial for achieving therapeutic efficacies. For example: dense distal accumulation and retention of lipiodol has been correlated with greater and longer-lasting necrosis controlling hepatocellular carcinoma [4, 5]. Successful uterine artery embolization (UAE) of symptomatic fibroids depends upon maximum infarction of the fibroids [6]. In an early comparative study for uterine artery embolization, spherical Polyvinyl alcohol (PVA) particles were considered inferior to similarly sized tris-acryl gelatin microspheres because of inadequate infarction [7]. Years later, in a randomized controlled study, embolics were shown equivalent; different embolics simply required different intraprocedural endpoints to achieve similar outcomes [8]. In both studies, technique of delivery was not specified. Thus, techniques to improve and standardize embolic delivery and intraprocedural endpoints are essential, though incompletely considered.

The purpose or primary outcome of this study is to objectively quantify the differences in distal target vessel penetration and overall total embolic delivered between continuous vs pulsed injection techniques using radiopaque microspheres evaluated with MicroCT imaging. These parameters were selected to directly reflect procedural performance and embolization efficacy. Secondary outcomes included correlation of MicroCT findings with histological analysis (HA) and feasibility of relying on MicroCT imaging alone for particle quantification.

## Materials and methods

### Animals

This prospective study was approved by the Animal Institutional Review Ethics Board. Seven male pigs weighing 35–40 kg were studied [9]. Ultrasound (Sonosite LX: FujiFilm, Bothell, USA) was performed immediately before the embolization to acquire kidney volumes (ellipsoid method) and assure no renal lesions were present. Measurements were performed pre-embolization and kidney explantation to avoid potential unforeseen changes in renal volume. The study was conducted from February 2022 to March 2025.

### Embolic material

Seventy-five-to-one-hundred-micron diameter radio-opaque DC Lumi Beads® (Boston Scientific, Natick, MA) were used to allow post-processing MicroCT scan imaging organ harvesting. Microspheres were prepared for injection as per manufacturer’s instructions. Spheres were mixed with 20 ml of contrast (Omnipaque 350; GE Healthcare, Mississauga, Canada) to achieve a uniform suspension and were delivered to the kidney using a 10 ml syringe. Two interventional radiologists with more than 5 and 25 years of experience in embolization procedures performed the injections. The operator who was not performing the embolization monitored technique and timing.

### Procedure

**Fig. 1:**
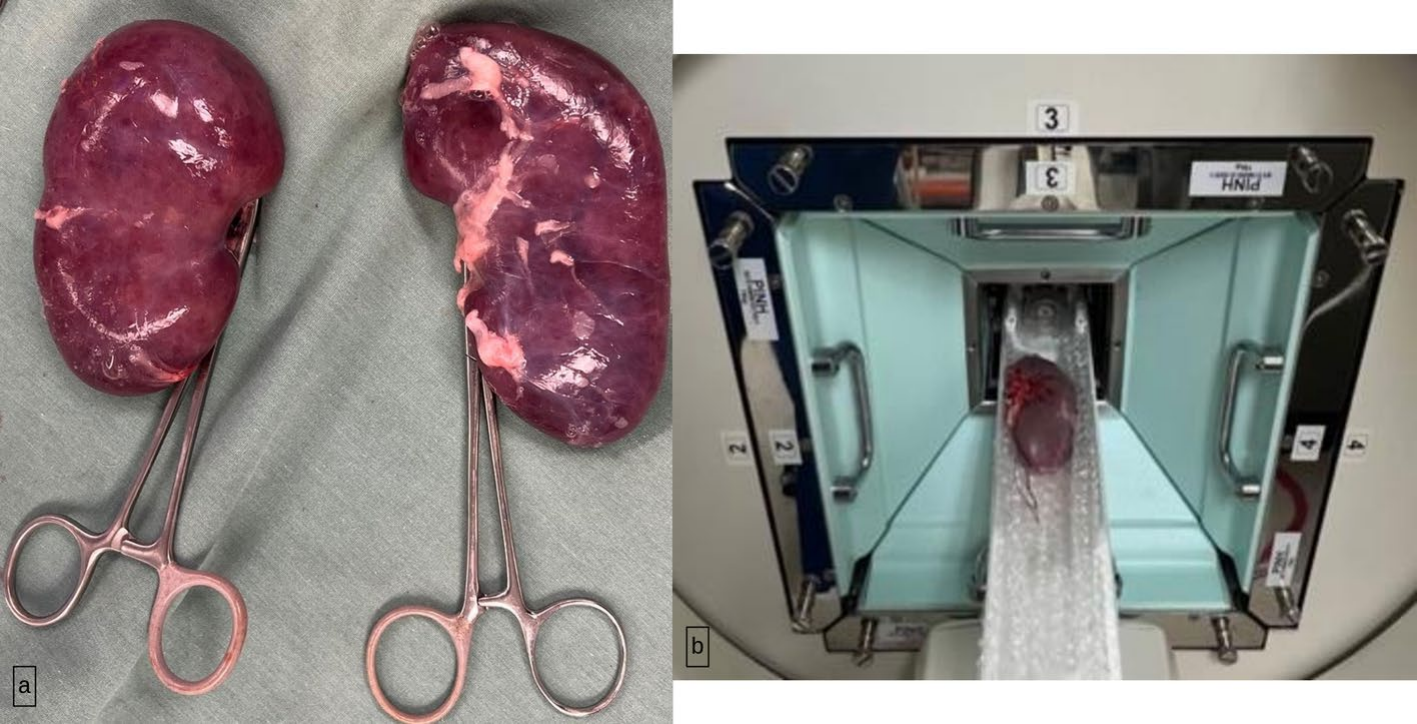
Intraprocedural angiographic image (renal embolization)

Veterinarians provided general anesthesia using ketamine and midazolam IV as premedication, and isoflurane and propofol for induction. Both kidneys were embolized in each subject, allowing each pig to act as its own control. Using a femoral approach, each renal artery was selected 1 cm beyond its origin with a 5Fr C2 catheter (Cook Medical, Bloomington, USA), followed by hand-injection angiography (60:40 contrast to saline). After randomization, one kidney was embolized using a continuous technique, the other with a pulsed technique. In the continuous group, embolics were injected by hand at 1 ml/sec until main renal artery stasis. In the pulsed group, 1 ml was injected every second with a 1-s pause, timed with a stopwatch, and stopped at stasis (Fig. 1). Hand injection was chosen over a pump to reflect common global practice.

**Fig. 2:**
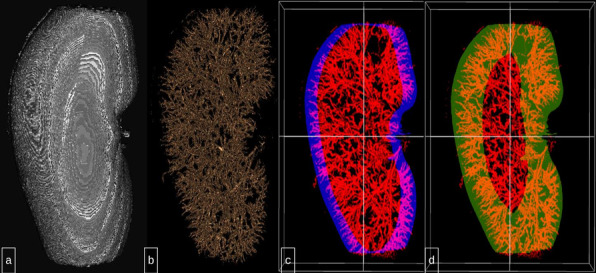
**a** Kidneys after being harvest. Renal hilum was clampled to prevent bleeding. **b** Kidney placed within the MicroCT machine

After embolization, subjects were euthanized immediately using potassium chloride IV. Both kidneys were harvested by veterinary staff using ligation of the renal hilum (Fig. 2). The hilum was examined upon explantation to verify no variations in vascular anatomy (e.g., Accessory renal arteries). To reduce CT artifacts from residual urine in the collecting system, 20 cc of saline was hand injected into the renal pelvis with an 18 g IV catheter. Kidneys were wrapped in saline-soaked gauze and immediately transferred for MicroCT scan assessment. After imaging, these were suspended in formaldehyde solution and transferred to pathology lab for analysis.

### Imaging and post-processing

The imaging equipment included: MicroCT (Precision X-Ray, North Branford, CT, USA) with spatial resolution of 100 microns, 225 kVp x-ray tube; and a flat panel amorphous silicon detector on a rotational C-arm gantry for cone-beam CT imaging (Fig. 2). Image quality, voxel noise, modulation transfer, CT number accuracy, and geometric accuracy characteristics were assessed using a water cylinder and MicroCT test phantoms. Images were acquired at 80kVp, 1.5 mA, and 0.1 mm resolution.

**Fig. 3:**
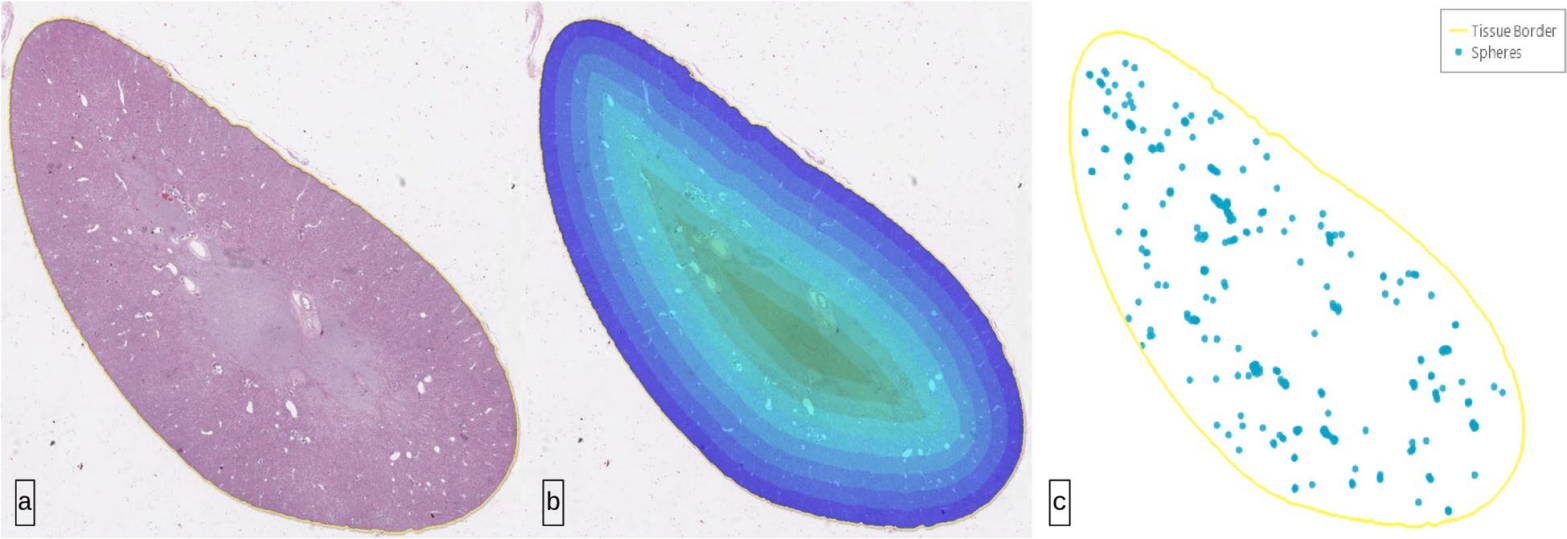
Mask volume renders, 50 and 100 pixel mask obtained with MicroCT scan

Arteries containing the radiopaque embolic were segmented with Ilastik 1.3.3post3 software [10]. With segmentation, renal parenchyma was excluded. Segmented vessels were overlaid with masks stratifying the kidney to cortical and distal regions for quantification of CT contrast using voxels. Masks were generated in MATLAB R2021a software. Kidney volumes were segmented from CT images and eroded by 50 pixels or 100 pixels (default setting of the software) from the surface of the kidney inwards, based on the size of the cortical portion to assess the embolic agent concentration (Fig. 3). The eroded kidney volumes or masks were used to subtract the whole kidney volumes to generate additional masks that stratified the kidney into cortical (distal) and non-cortical regions.

CT quantification of findings was performed by overlaying cortical masks to segmented vessels. Pixel values of both volumes were binary (where positive and negative images were expressed in terms of 0 and 1, as per computer analysis), thus the intersection of masks and vessels at positive integers indicated CT presence of contrast agents in the specified region. The total number of intersecting points were divided by the respective kidney volume to normalize the CT signal and obtain an embolization efficiency.

### Histological analysis

**Fig. 4:**
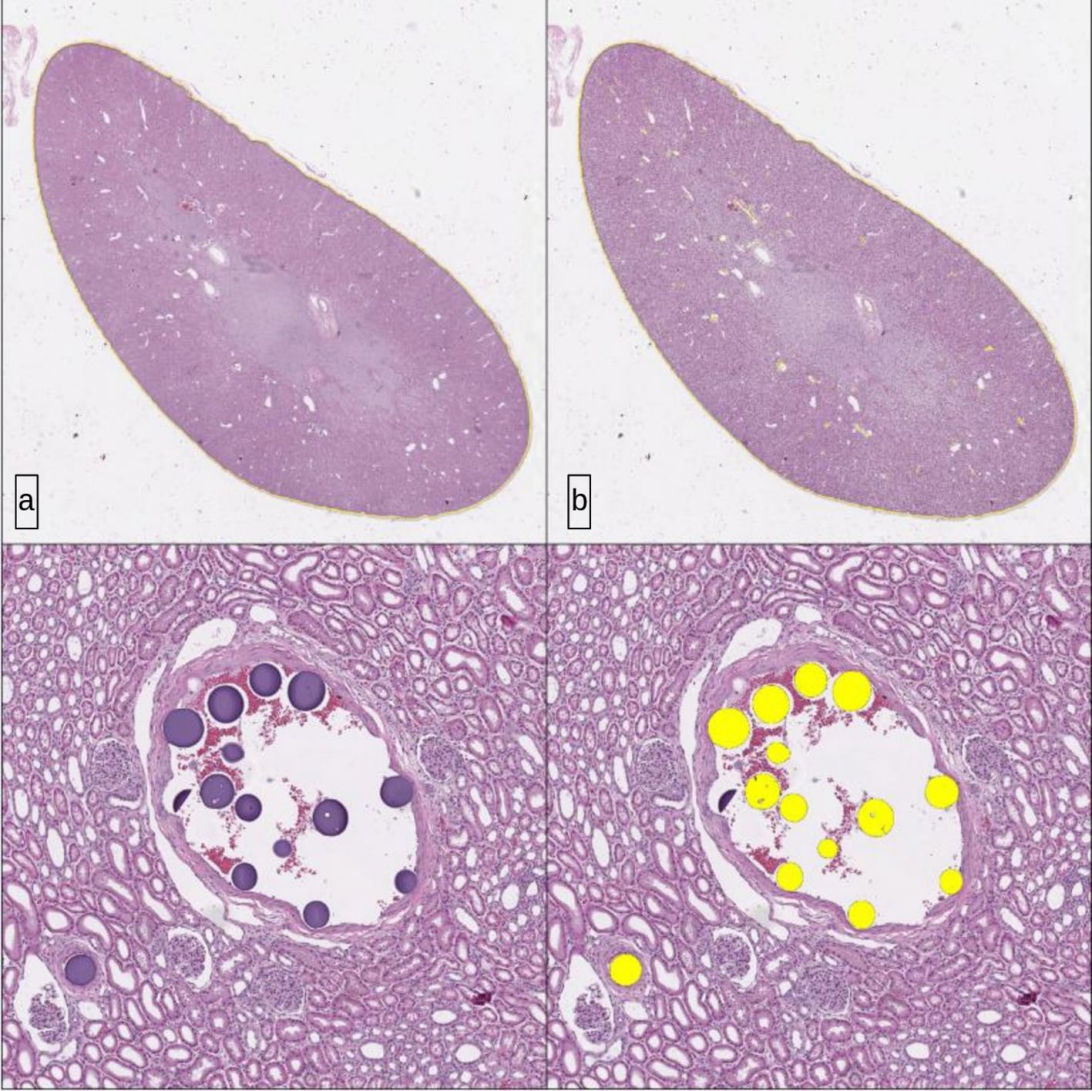
a) H&E histological analysis. **b** Masks obtained to emulate evaluation given pixels from MicroCT (distance from surface inwards. **c** Microscopic distribution of particles in renal cortex

Kidney samples were fixed overnight in 10% neutral buffered formalin and grossly sectioned in longitudinal plain into 7 equal slices. These slices were dehydrated and infiltrated with paraffin in a tissue processor (Sakura Histo-Tek VP1) with 14 h programmed cycle. Thereafter, the slices were embedded in paraffin and sectioned longitudinally on the microtome (Thermo Shandon Finesse ME +) in 4um sections. The resulting microtome sections were stained with hematoxylin & eosin (Harris Hematoxylin (Stat Lab: SL90-1) and Eosin (Leica Surgipath: 3,801,600). Histological analysis was performed on the initial two animals (four kidneys) to ascertain if MicroCT findings were comparable, after which MicroCT was used exclusively. The scanner was an Aperio AT2, the objective was a UPlanS Apo 20x/0.75 NA, and pixel resolution was 0.5 um/pixel. Analysis was performed using Indica Labs' HALO software, with the Vacuole analysis package (Fig. 4).

### Statistical analysis

T-test and Wilcoxon tests were used to determine statistical significance. RSTudio (Posit, Vienna, Austria) was used to graph and compare results. Results for continuous variables are presented with range and median. As this study is exploratory with no prior relevant studies published, an appropriate sample could not be determined, and the study is likely not powered to detect small-to-moderate differences in outcomes.

## Results

Seven pigs and 14 kidneys were treated as follows: in the odd numbered subjects the left kidney was embolized with the pulsed technique and the right kidney with the continuous technique. In even pairs the left kidney was embolized with the continuous technique and the right with pulsed. All procedures were technically successful (defined as completion of embolization upon fluoroscopic visualization of stasis in the main renal artery beyond the catheter tip). No anatomical variants or accessory arteries were present.

The median renal volume was 111.4 ml (46—138 ml). The median renal volume in the continuous and pulsed groups was 78.1 ml (49—138 ml) and 81 ml (46—111 ml), respectively. The median injected embolic volumes were 14 ml (13—20 ml), continuous group; and 13.1 ml (8—22 ml) in the pulsed group (Table 1).

**Table 1:** Kidney size and embolic agent volume

Parameter	Continuous	Pulsed	p-value
Median Renal Volume (ml)	78.1 (49–138)	81.0 (46–111)	N/A
Median Embolic Volume Injected (ml)	14.0 (12–20)	13.1 (8–22)	N/A
Median Embolic Load (particles)	6,806	6,174	N/A
% Embolized Cortex at 50 pixels	1.1% (0.1–2.3)	0.7% (0.5–1.1)	0.3
% Embolized Cortex at 100 pixels	2.9% (1.3–4.2)	2.3% (1.3–3.2)	0.3
Embolic Load/Renal Volume Ratio	0.20	0.16	0.2

Following embolization and kidney retrieval from the initial two animals, renal specimens were sectioned and analyzed histologically to quantify intra-cortical particle distribution. Sectioning thickness was standardized to match MicroCT analysis (50 and 100 pixels from the cortical surface) to allow for direct comparison between modalities. Quantitative analysis demonstrated consistent particle concentrations between histopathological assessment and MicroCT imaging. Across both animals, the continuous delivery technique consistently resulted in higher particle densities compared to the pulsed method.

In the first subject, histological analysis determined that continuous delivery yielded a total of 3,688 particles, compared to 3,049 with pulsed. This increased particle load was achieved with a smaller renal volume (75 mL vs. 138 mL). MicroCT analysis at the 50-micron mask quantified 3,523 particles in the continuous group versus 2,923 particles with pulsed, while at the 100-micron mask 165 particles were identified with continuous compared to 126 with pulsed. In the second subject, histological analysis showed comparable total particle counts between delivery methods (3,118 continuous vs. 3,125 pulsed), continuous delivery was associated with a slightly smaller renal volume (85 mL vs. 93 mL). MicroCT evaluation at the 50-micron mask demonstrated 2,986 particles with continuous delivery versus 2,884 particles with pulsed; at the 100-micron mask, particle counts were 132 and 241, respectively (Table 1).

**Fig. 5:**
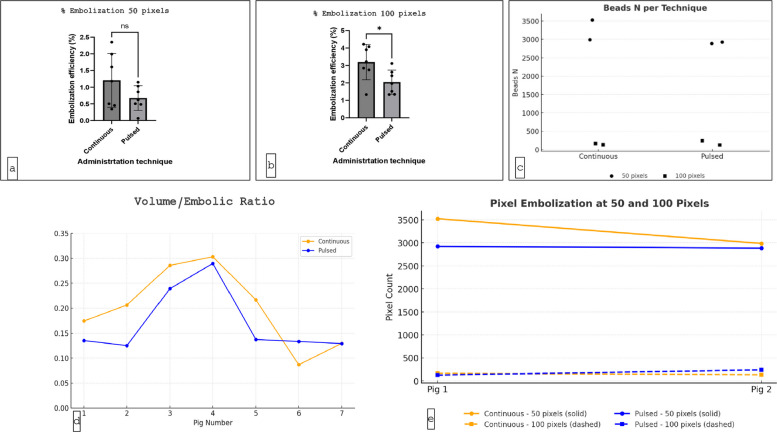
Left-sided kidney (same kidney): **a** MicroCT evaluation, **b** vessel mapping, **c** H&E histological evaluation, **d** Microscopic distribution of particles in renal cortex

**Fig. 6:**
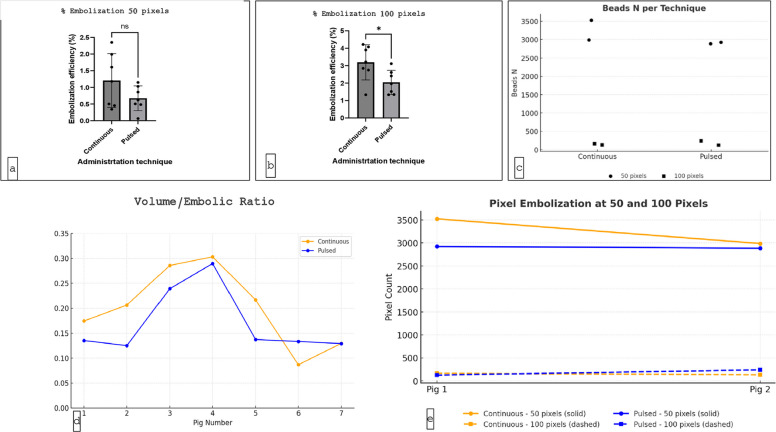
Percentage embolization at 50 pixels (**a**) and 100 pixels (**b**) for mL. **C** Representation of number of beads comparing both techniques at 50 and 100 pixels. **D** Trend volume/embolic ratio with MicroCT scan. E) Trend N of beads at 50 and 100 pixels comparing both delivery techniques

The median percentage of the embolized renal cortex, using the Mask 50 pixels from the renal surface, was 1.1% (0.1–2.3%) with the continuous technique, compared to 0.7% (0.5–1.1%) with the pulsed (p = 0.3). The median percentage of embolized renal cortex, using the Mask 100 pixels from the renal surface, was 2.9% (1.3–4.2%) with the continuous technique, compared to 2.3% (1.3–3.2%) with the pulsed (p = 0.3) (Fig. 5). Overall, the volume of embolic agent injected over the volume of kidney parenchyma ratio with the continuous embolization technique was 0.20 vs 0.16 in the pulsed group (p = 0.2) demonstrating a larger but not statistically significant concentration of embolic agent in the renal parenchyma. In each case, the continuous technique resulted in a greater absolute concentration of embolic (at 50- and 100-pixel levels) compared to pulsed technique (Fig. 6 and Table 1).

## Discussion

The continuous embolization technique resulted in higher concentrations of embolic agent in the distal vessels in this in-vivo experiment compared to the pulsed technique, as well as greater volumes, though there was no significant difference observed. Nevertheless, the results suggest that further study is needed as embolic technique may have potential applicability in daily interventions, be they transarterial therapy of tumors, prostatic or uterine embolization or other settings where denser packing and delivery of embolics may yield better therapeutic outcomes [4, 5, 11, 12].

Despite advances in research, manufacturing, and application of embolics, there has been little to no investigation into the effect of differing embolic techniques on the extent and efficacy of occlusion. In a rabbit study by Choe et al. [13], different concentrations of PVA were delivered at different rates by continuous hand injection. Distal embolization was angiographically superior with a slower infusion of more diluted particles. The impact of pulsed delivery was not assessed. A recent review article by Talaie et al. examining factors that affect arterial embolization discussed the hemodynamics influencing embolization but did not address potential differences between continuous vs pulsed [14]. Similarly, embolization literature does not specify the precise embolization technique used. One study comparing non-spherical polyvinyl alcohol particles versus tris-acryl microspheres found no difference in dominant fibroid necrosis [8]. The technique of embolization was not described. The lack of difference may reflect similarities in particle effect, or the possibility of overlooked differences related to particle specific delivery techniques and embolization endpoints.

Multiple studies have highlighted the importance of consistent distal penetration of an embolic upon durable clinical efficacy. Carnevale et al. demonstrated that a larger overall volume of embolic delivered distally into the prostate led to greater coagulative necrosis and prostate shrinkage [11]. Similarly, the efficacy and safety of bladder embolization for treatment of intractable hematuria depended on distal penetration of embolics [15]. Preoperative distal particle embolization of intracranial meningiomas was associated with significantly less intra-operative blood loss [16]. The technique of delivery may influence outcomes, particularly in interventional oncology. Thus, distal penetration of embolics is often desired and required for therapeutic benefit.

Swine porcine models have been used due to their anatomical and physiological similarity to human vascular anatomy and pathologies such as tumors [17–20]. Using the LUMI radio-opaque microspheres allowed us to use MicroCT to assess the results of each technique and quantify outcomes of the entire kidney instead of using non-radio-opaque embolic agents and performing a limited histological assessment of parts of the kidney. While feasibility of LUMI bead imaging using MicroCT has been previously reported [20], its use for assessing the microvascular distribution and delivery techniques is novel. After demonstrating concordance between MicroCT and histology in the initial specimens, we adopted MicroCT exclusively to quantify embolic distribution in the remaining cohort, leveraging its non-destructive full-organ analysis capability. Unlike histopathological analysis, which relies on limited tissue sampling and introduces potential sectioning artifacts, MicroCT provided high-resolution, full-organ 3D quantification of embolic distribution. This facilitated comprehensive and objective comparisons of embolization techniques. Furthermore, these results comparing imaging with histological analysis with a random assignment and paired-organ control have not been assessed previously.

This study has several limitations. These include the small number of test subjects, and the single type and size of embolic and anatomic vascular territory used. Our results might not apply to other spherical or non-spherical embolics, or liquids. Also, variations in pulse frequency and volume of particles delivered per pulse may impact hemodynamic effects yielding a different outcome. Extrapolation from more distal and denser embolization in the porcine renal model might not reflect findings in humans. A broader study, with varied agents might modify or amplify these preliminary findings, including different brands and sizes of embolics, as differences in shape, conformability, surface charge, thrombogenicity and other features may magnify potential outcome differences. Also, an objective endpoint for termination of delivery of particle embolic has not been determined beyond perception or visualization of reflux in clinical practice. This can be considered a critique of the study despite this being current clinical practice. However, an objective of the study was also to assess volume of embolic delivered distally. This would not be influenced by the endpoint of the embolic delivery (visualized reflux).

A statistically significant difference in embolic concentration at 50 and 100 pixels between the two methods was not identified. However, overall results with continuous embolization exceeded pulsed technique, and an absolute consistent difference was seen between techniques. As this was a pilot study, with an arbitrarily chosen number of subjects, determining sample size to identify or exclude differences was not possible prospectively.

## Conclusion

The continuous technique for transarterial embolization resulted in a higher particle embolic agent concentration, although not statistically significant, within the distal porcine renal tissue and within the entire kidney when compared with the traditional pulsed technique in an in-vivo model using radio-opaque microspheres as the embolic agent. The findings suggest that the injection technique for particle embolization can impact the degree of embolic delivered.

## Data Availability

Yes.

## Abbreviations

IV: Intravenous
CT: Computed Tomography
MicroCT: Micro Computed Tomography
PVA: Polyvinyl Alcohol
HA: Histological Analysis
UAE: Uterine Artery Embolization
